# Supplementary material for: Single-molecule observation of ATP-independent SSB displacement by RecO in Deinococcus radiodurans
Source: eLife. 2020 Apr 16;9:e50945. doi: 10.7554/eLife.50945 (PMC7200156; doi:10.7554/eLife.50945)
Supplement: Figure 2—source data 1. [file elife-50945-fig2-data1.docx]

Figure 2––Source data 1. Data summary table for the results shown in figure 2E.

| RecO concentration (µM) | On-rate of dT70  (s^-1^) | On-rate of drSSB-dT70  (s^-1^) |
| --- | --- | --- |
| 0.1 | 0.0168 | 0.00264 |
| 0.33 | 0.0231 | 0.00607 |
| 1 | 0.0885 | 0.00989 |
| 3 | N/A | 0.0251 |
