# Supplementary material for: Single-molecule observation of ATP-independent SSB displacement by RecO in Deinococcus radiodurans
Source: eLife. 2020 Apr 16;9:e50945. doi: 10.7554/eLife.50945 (PMC7200156; doi:10.7554/eLife.50945)
Supplement: Figure 2—source data 2. [file elife-50945-fig2-data2.docx]

Figure 2––Source data 2. Data summary table for the results shown in Figure 2G.

| drRecO concentration (µM) | Fraction of No RecO binding  (%) | Fraction of RecO binding  (%) |
| --- | --- | --- |
| 0 | 99.5 | 0.5 |
| 0.1 | 71.8 | 28.2 |
| 0.33 | 52.3 | 47.7 |
| 1 | 9.1 | 90.9 |
| 3 | 5.1 | 94.9 |
