# Supplementary material for: Single-molecule observation of ATP-independent SSB displacement by RecO in Deinococcus radiodurans
Source: eLife. 2020 Apr 16;9:e50945. doi: 10.7554/eLife.50945 (PMC7200156; doi:10.7554/eLife.50945)
Supplement: Figure 4—source data 1. [file elife-50945-fig4-data1.docx]

Figure 4––Source data. Data summary table for the results shown in Figure 4D.

| drRecO concentration (µM) | Fraction of Colocalization  (%) | Fraction of Dissociation (%) |
| --- | --- | --- |
| 0 | 72.0 | 28.0 |
| 0.1 | 35.9 | 64.1 |
| 0.33 | 11.0 | 89.0 |
| 1 | 2.4 | 97.6 |
| 3 | 5.9 | 94.1 |
