# Supplementary material for: Single-molecule observation of ATP-independent SSB displacement by RecO in Deinococcus radiodurans
Source: eLife. 2020 Apr 16;9:e50945. doi: 10.7554/eLife.50945 (PMC7200156; doi:10.7554/eLife.50945)
Supplement: Figure 6—source data 1. [file elife-50945-fig6-data1.docx]

Figure 6––Source data 1. Data summary table for the results shown in Figure 6B.

|  | *ka* (s^-1^) | standard deviation of *k_a_* |
| --- | --- | --- |
| dT40 | 0.021 | 0.002 |
| dT50 | 0.909 | 0.002 |
| dT60 | 0.105 | 0.001 |
| dT70 | 0.111 | 0.001 |
