# Supplementary material for: Single-molecule observation of ATP-independent SSB displacement by RecO in Deinococcus radiodurans
Source: eLife. 2020 Apr 16;9:e50945. doi: 10.7554/eLife.50945 (PMC7200156; doi:10.7554/eLife.50945)
Supplement: Figure 6—source data 2. [file elife-50945-fig6-data2.docx]

Figure 6––Source data 2. Data summary table for the results shown in Figure 6C.

|  | Single transition (%) | Double transition (%) |
| --- | --- | --- |
| dT40 | 93.1 | 6.9 |
| dT50 | 96.1 | 3.9 |
| dT60 | 98.2 | 1.8 |
| dT70 | 99.6 | 0.4 |
