# Supplementary material for: Single-molecule observation of ATP-independent SSB displacement by RecO in Deinococcus radiodurans
Source: eLife. 2020 Apr 16;9:e50945. doi: 10.7554/eLife.50945 (PMC7200156; doi:10.7554/eLife.50945)
Supplement: Figure 6—source data 3. [file elife-50945-fig6-data3.docx]

Figure 6––Source data 3. Data summary table for the results shown in Figure 6D.

| drRecO  concentration  (µM) | *k_a1_*  (s^-1^) | standard deviation of *k_a1_* | *k_a2_*  (s^-1^) | standard deviation of *k_a2_* |
| --- | --- | --- | --- | --- |
| 0.2 | 0.00395 | 0.00192 | 0.0273 | 0.0129 |
| 1 | 0.0208 | 0.00199 | 0.0300 | 0.0060 |
| 5 | 0.0434 | 0.00134 | 0.0288 | 0.0098 |
