# Supplementary material for: Single-molecule observation of ATP-independent SSB displacement by RecO in Deinococcus radiodurans
Source: eLife. 2020 Apr 16;9:e50945. doi: 10.7554/eLife.50945 (PMC7200156; doi:10.7554/eLife.50945)
Supplement: Figure 8—source data 1. [file elife-50945-fig8-data1.docx]

Figure 8––Source data. Data summary table for the results shown in Figure 8C.

|  | Single transition (%) | Double transition (%) | Triple transition (%) | >Quardruple transition (%) |
| --- | --- | --- | --- | --- |
| drSSB-dT70 | 98.0 | 2.0 | 0.0 | 0.0 |
| dT40 | 33.8 | 43.3 | 16.6 | 6.3 |
| dT50 | 89.5 | 9.7 | 0.8 | 0 |
| dT60 | 90.5 | 8.8 | 0.7 | 0 |
| dT70 | 98.7 | 1.2 | 0 | 0.1 |
