# Supplementary material for: Single-molecule observation of ATP-independent SSB displacement by RecO in Deinococcus radiodurans
Source: eLife. 2020 Apr 16;9:e50945. doi: 10.7554/eLife.50945 (PMC7200156; doi:10.7554/eLife.50945)
Supplement: Figure 9—source data 1. [file elife-50945-fig9-data1.docx]

Figure 9––Source data. Data summary table for the results shown in figure 9C.

|  | drRecO binding with intermediate (%) | drRecO binding  without intermediate (%) | No drRecO binding (%) |
| --- | --- | --- | --- |
| wt-drRecO | 47.0 | 52.4 | 0.6 |
| K35E/R39E drRecO | 78.2 | 13.0 | 8.8 |
| R195E/R196E drRecO | 91.1 | 4.3 | 4.6 |
